# Cause-specific mortality in adult epilepsy patients from Tyrol, Austria: hospital-based study

**DOI:** 10.1007/s00415-014-7536-z

**Published:** 2014-10-26

**Authors:** Claudia A. Granbichler, Willi Oberaigner, Giorgi Kuchukhidze, Gerhard Bauer, Jean-Pierre Ndayisaba, Klaus Seppi, Eugen Trinka

**Affiliations:** 1 Department for Neurology, Medical University Innsbruck, Innsbruck, Austria; 2 Institute for Clinical Epidemiology, TILAK, Wilhelm-Greil-Str. 25, 6020 Innsbruck, Austria; 3 Department of Public Health Technology Assessment, UMIT, University for Health Sciences, Medical Informatics and Technology, Eduard Wallnöfer-Zentrum 1, 6060 Hall in Tirol, Austria; 4 Department for Neurology Christian Doppler Medical Center, Paracelsus Medical University, Ignaz Harrer Str. 79, 5020 Salzburg, Austria

**Keywords:** Epilepsy, Epidemiology, Mortality, Cause specific SMR, Causes of death

## Abstract

Epilepsy is a devastating condition with a considerable increase in mortality compared to the general population. Few studies have focused on cause-specific mortality which we analyse in detail in over 4,000 well-characterized epilepsy patients. The cohort comprised of epilepsy patients ≥18, treated between 1970 and 2009 at the epilepsy clinic of Innsbruck Medical University, Austria, and living in the province of Tyrol, Austria. Epilepsy diagnosis was based on ILAE guidelines (1989); patients with brain tumor were excluded. Deceased patients and causes of death (ICD-codes) were obtained via record linkage to the national death registry. We computed age-, sex-, and period-adjusted standardized mortality rates (SMR) for 36 diagnoses subgroups in four major groups. Additional analyses were performed for an incidence cohort. Overall cohort: 4,295 patients, 60,649.1 person-years, 822 deaths, overall SMR 1.7 (95 % CI 1.6–1.9), highest elevated cause-specific SMR: congenital anomalies [7.1 (95 % CI 2.3–16.6)], suicide [4.2 (95 % CI 2.0–8.1)], alcohol dependence syndrome [3.9 (95 % CI 1.8–7.4)], malignant neoplasm of esophagus [3.1 (95 % CI 1.2–6.4)], pneumonia [2.7 (95 % CI 1.6–4.2)]. Incidence cohort: 1,299 patients, 14,215.4 person-years, 267 deaths, overall SMR 1.8 (95 % CI 1.6–2.1), highest elevated cause-specific SMR congenital anomalies [10.8 (95 % CI 1.3–39.3)], suicide [6.8 (95 % CI 1.4–19.8)], alcohol dependence syndrome (6.4 [95 % CI 1.8–16.5)], pneumonia [3.9 (95 % CI 1.8–7.4)], cerebrovascular disease at 3.5 (95 % CI 2.6–4.6). Mortality due to mental health problems, such as suicide or alcohol dependence syndrome, malignant neoplasms, and cerebrovascular diseases was highly increased in our study. In addition to aim for seizure freedom, we suggest improving general health promotion, including cessation of smoking, lowering of alcohol intake, and reduction of weight as well as early identification of psychiatric comorbidity in patients with epilepsy.

## Introduction

Epilepsy is a potentially life-threatening condition with a considerable increase in mortality compared to the general population. Depending on the population under study these numbers vary substantially and are highest for difficult to treat and epilepsy surgery patients and lowest in population-based studies [12, 34].

Early investigations of causes of death in the epilepsy population have been conducted since the 1970s where underlying disorders causing epilepsy were the most frequently recorded causes of death, especially during the first years following diagnosis. Investigations were sometimes inconclusive and findings from some studies could not be confirmed by others. Furthermore, differences in methodology and study population make comparisons between cohorts difficult [34].

In the present study, we report the overall and cause-specific mortality in a large hospital-based epilepsy population from a neurological service in Innsbruck, Austria. Data from this cohort were previously described [37] and we now extended observation with an additional 10 year follow-up period to up to 39 years. We report on current overall mortality statistics and present SMRs on causes of death compared to the general population of the same geographic area. Main features of this large cohort are its epilepsy diagnosis according to ILAE guidelines [10] and long duration of follow-up.

## Methods

At the epilepsy outpatient clinic at the University Hospital for Neurology in Innsbruck, Austria, which is the only specialized epilepsy outpatient clinic in the province of Tyrol with its over 700,000 inhabitants [35], patients were recorded in a database as early as the 1970 by one specialist neurologist (GB). Around the turn of the millennium the original database was transferred and upgraded, and additions of supplementary clinical data made (ET, J-PN, CG). Patient data were updated at every visit to the outpatient department. Parts of this cohort have already been described previously [37]. During 2008 and 2009 all patient records were reviewed (CG) for accuracy of the entries and missing data added where available. The present study included all patients with a diagnosis of epilepsy according to ILAE criteria [10] who visited the epilepsy outpatient clinic between January 1st, 1970 and December 31st, 2009, were above 18 years of age at end of study and permanent residents of Tyrol, Austria. Patients with a brain tumor as suspected cause of epilepsy were excluded from the study. Follow-up was terminated death or on December 31st, 2009, whichever occurred first.

The database was linked to the national death registry, applying a probabilistic record linkage method [30], to identify deceased patients and their causes of death as stated on death certificates via ICD-9 or ICD-10 codes. In Austria only one ICD-code is entered into the death registry by the reporting physician as cause of death, possible contributing causes cannot be recorded. Demographic statistics were computed and standardized mortality rates (SMR) calculated in comparison to the general population of the same province adjusted for age, sex, and period of death-year. Causes of death were grouped into four major groups and 36 subgroups (Table 1). This grouping-system was based on the one published by Nilsson et al. [29].

**Table 1:** Causes of death-groups according to ICD code

Cause of death	ICD-9	ICD-10
Malignant neoplasms		
Malignant neoplasms overall	140–208	C00–C97
Malignant neoplasms except brain tumor	140–208 except 191	C00–C97 except C69–C72
Of brain and other parts of nervous system	191–192	C69–C72
Of oral cavity and pharynx	140–149	C00–C14
Of esophagus	150	C15
Of liver and intrahepatic bile ducts	155	C22
Of respiratory and intrathoracic organs	160–165	C30–C39
Of bone, connective tissue, skin and breast	170–175	C40, C41, C45–C49, C43–C44
Malignant melanoma	172	C43–C44
Of female breast	174	C50
Of lymphatic and hematopoietic tissue	200–208	C81–C96
Lymphosarcoma and reticulosarcoma	200	C85, C83.3
Hodgkin’s disease	201	C81
Other malignant neoplasms of lymphoid and histiocytic tissue	202	C96
Psychiatric diseases		
Mental disorders	290–319	F00–F99
Dementia	NA	F00–F02
Senile dementia	290.0	NA
Presenile dementia	290.1	NA
Alcoholic psychoses	291	F10.5
Alcohol dependence syndrome	303	F10.2
Drug dependence	304	F11.2, F12.2, F13.2, F14.2, F15.2, F16.2, F17.2, F18.2, F19.2
Organ diseases		
Diseases of inner organs	309–578, 740–759	I00–I99, J00–J99, K00–K93, Q00–Q99
Diseases of the circulatory system	390–459	I00–I99
Ischemic heart disease	410–414	I20–I25
Cerebrovascular disease	430–438	I60–I69
Diseases of the respiratory system	460–519	J00–J99
Pneumonia	480–486	J12–J18
Chronic obstructive pulmonary disease, emphysema, asthma	490–493	J40–J47
Diseases of the digestive system	520–579	K00–K93
Other diseases of the digestive system	570–578	K90–K93
Congenital anomalies	740–759	Q00–Q99
External causes		
External causes overall	800–999	S00–T98, U11, U31
Results from injuries	NA	T90–T94
Transport accidents	800–849	U11
Accidental falls	880–888	NA
Accidents caused by fire and flames	890–899	T20–T32
Accidents caused by submersion, suffocation, and foreign bodies	910–915	T71, T75.1
Suicide	950–959	U31
Injury undetermined whether accidentally or purposely inflicted	980–989	NA
Injury not specified	NA	T14.9

*NA* not applicable

In addition, if an epilepsy diagnosis was established within 365 days following an individual’s first ever epileptic seizure, they were considered an incidence case. For these cases additional mortality-analyses was performed as the incidence cohort. Stata 2012 for Windows (Stata Inc. 2012) was used for all statistical calculations.

## Results

Demographic data of overall and incidence cohort are presented in Table 2.

**Table 2:** Overview of demographic data

	Overall cohort	Incidence cohort
*N* (F/M)	4,295 (2,037/2,258)	1,299 (573/726)
Age at seizure onset	29.6 (0–99.4)	44.3 (0.3–99.4)
Age at study entry	38.8 (0.4–99.4)	44.3 (0.4–99.4)
Age at study exit	46.3 (18.0–99.4)	50.3 (18.0–9.4)
Epilepsy duration	23.3 (0–93.0)	11.0 (0–40.8)
Person-years (F/M)	60,649.1	14,215.4
Deaths	822	267

*N* number of subjects, *F* female patients, *M* male patients

### Overall cohort

Overall SMR for the cohort was 1.7 (95 % CI 1.6–1.9), 1.8 (95 % CI 1.6–2.0) for women, 1.7 (95 % CI 1.5–1.9) for men. Cause-specific SMR values for the overall cohort are presented in Table 3.

**Table 3:** Standardized mortality rates in the overall cohort by cause of death-groups

Cause of death	Observed deaths	Expected deaths	SMR	95 % CI
Malignant neoplasms				
Malignant neoplasms overall	163	134	1.2*	1.0–1.4
Malignant neoplasms except brain tumor	158	133	1.2*	1.0–1.4
Of brain and other parts of nervous system	5	3	**1.5**	0.5–3.4
Of oral cavity and pharynx	6	3	**1.8**	0.7–3.9
Of esophagus	7	2	3.1*	1.2–6.4
Of liver and intrahepatic bile ducts	7	4	**1.6**	0.7–3.3
Of respiratory and intrathoracic organs	44	30	1.5*	1.1–2.0
Of bone, connective tissue, skin and breast	0	0	**0**	0.0–8.7
Malignant melanoma	3	3	**1.2**	0.2–3.5
Of female breast	10	10	**1.0**	0.5–1.9
Of lymphatic and hematopoietic tissue	14	10	**1.4**	0.8–2.3
Lymphosarcoma and reticulosarcoma	5	3	**1.8**	0.6–4.1
Hodgkin’s disease	1	0	**2.9**	0.1–16.0
Other malignant neoplasms of lymphoid and histiocytic tissue	0	2	**0**	0.0–2.5
Psychiatric diseases				
Mental disorders	11	4	2.8**	1.4–4.9
Dementia	0	0	**0**	0.0–42.8
Senile dementia	0	0	**0**	0.0–88.0
Presenile dementia	0	0	**0**	0.0–811.2
Alcoholic psychoses	0	0	**0**	0.0–73.8
Alcohol dependence syndrome	9	2	3.9**	1.8–7.4
Drug dependence	0	1	**0**	0.0–3.6
Organ diseases				
Diseases of inner organs	431	252	1.7***	1.6–1.9
Diseases of the circulatory system	350	204	1.7***	1.5–1.9
Ischemic heart disease	150	96	1.6***	1.3–1.8
Cerebrovascular disease	113	44	2.6***	2.1–3.1
Diseases of the respiratory system	53	28	1.9***	1.4–2.5
Pneumonia	18	7	2.7***	1.6–4.2
Chronic obstructive pulmonary disease, emphysema, asthma	18	15	**1.2**	0.7–1.9
Diseases of the digestive system	23	20	**1.1**	0.7–1.7
Other diseases of the digestive system	7	9	**0.8**	0.3–1.6
Congenital anomalies	5	1	7.1**	2.3–16.6
External causes				
External causes overall	78	39	2.0***	1.6–2.5
Results from injuries	2	0	**6.6**	0.8–23.8
Transport accidents	9	7	**1.3**	0.6–2.5
Accidental falls	0	0	**0**	0.0–24.0
Accidents caused by fire and flames	0	0	**0**	0.0–34.5
Accidents caused by submersion, suffocation, and foreign bodies	6	3	**2.0**	0.7–4.3
Suicide	9	2	4.2***	2.0–8.1
Injury undetermined whether accidentally or purposely inflicted	2	1	**2.5**	0.3–9.1
Injury not specified	1	0	**5.4**	0.1–30.4

* *p* < 0.05; ** *p* < 0.01; *** *p* < 0.001

### Incidence cohort

Overall mortality and cause specific mortality in the incidence cohort showed similar values as in the overall cohort with an overall SMR of 1.8 (95 % CI 1.6–2.1), 1.8 (95 % CI 1.5–2.2) for women, 1.8 (95 % CI 1.6–2.2) for men. Significantly elevated SMR values in the cause-specific evaluation were: congenital anomalies at 10.8 (95 % CI 1.3–39.3), suicide at 6.8 (95 % CI 1.4–19.8), alcohol dependence syndrome at 6.4 (95 % CI 1.8–16.5), mental disorders at 4.6 (95 % CI 1.5–10.8), pneumonia at 3.9 (95 % CI 1.8–7.4), cerebrovascular disease at 3.5 (95 % CI 2.6–4.6), diseases of the respiratory system at 2.5 (1.6–3.8), diseases of inner organs at 2.0 (95 % CI 1.7–2.3), diseases of the circulatory system at 2.0 (95 % CI 1.7–2.4), ischemic heart disease at 1.9 (95 % CI 1.4–2.4), and malignant neoplasms overall at 1.4 (95 % CI 1.0–1.8). Other subgroups did not show elevated SMRs or could not reach significance level.

## Discussion

In this mortality, study with over 60,000 person-years of follow-up we found an excess mortality of 1.7–1.8 times compared to the general population of the same region. The most highly increased causes of death were congenital anomalies, suicide, alcohol dependence syndrome, malignant neoplasms of the esophagus, pneumonia, and cerebrovascular disease.

A large, prospective long-term population-based cohort is considered gold standard for epidemiological research. Despite its good representation of the general population, it also has significant drawbacks, which are most importantly low accuracy of epilepsy diagnosis and limited numbers of patients ascertained. Some investigators classify subgroups of definite, probable and possible epilepsy but do not take any further measures to clarify uncertain cases [28]. But even hospital-based cohorts sometimes struggle with this problem: one study found erroneous epilepsy diagnoses in 21 % of patients [29]. Older studies carry the additional drawback of incomparable epilepsy classification systems that are not in use nowadays [16]. The main advantage of the present study is its large hospital-based cohort with an ongoing recruitment of 40 years and a reliable epilepsy diagnosis, based on the ILAE classification system of 1989 [10], as all patients were treated in one specialized epilepsy outpatient clinic. Furthermore, inpatient admission was not required for inclusion and patients could also be referred to the outpatient clinic by their family doctor or specialist physician, which we assume to lower the bias towards more severe epilepsy cases. It is common practice in urban regions of Austria to admit patients with a first epileptic seizure for inpatient neurologic evaluation of the event, followed by regular check-ups at the epilepsy outpatient clinic. As our service represents the only specialized epilepsy unit in Tyrol province, a high percentage of epilepsy patients from the region are expected to be seen there. Nevertheless, a certain selection-bias towards more severe epilepsy cases cannot be completely excluded.

Due to a lower mortality in the province of Tyrol compared to other provinces of Austria [35], mortality calculations were made in comparison to the general population of the province, thus, producing more reliable numbers and avoiding a potential bias seen in other studies, where patients were compared to a country’s overall population [26, 31]. Comparison was age-, sex-, and period of death-year adjusted as this may otherwise carry a significant bias, especially with longer follow-up.

Novel imaging techniques such as MRI or PET help establishing the etiology of epilepsy, including cerebral neoplasm. However, these methods have not yet been available during some of the earlier studies or their quality was that of contemporary standards. As shown in a population based study in Rochester, USA, there is no significant excess of malignancies when patients carrying a diagnosis of cancer prior to that of epilepsy were excluded from analysis [16]. As recruitment of patients in our study started as early as the 1970s such a bias may also have occurred for a fraction of our patients.

### Overall mortality

Overall mortality was elevated to an SMR of 1.7 (95 % CI 1.6–1.9) and 1.8 (95 % CI 1.6–2.1) for the incidence cohort. Depending on the population studied, SMRs in epilepsy patients were previously reported in a wide range from 1.6 to up to 15.9 in highly selected cohorts [34]. The highest mortality occurred during the first years following diagnosis and remained elevated up to 30 years after diagnosis [12, 16, 28, 29, 34, 37]. Some studies showed a late increase 20–25 years after initial diagnosis [34] which, in contrast, was not seen in our cohort. The comparatively lower mortality may be due to the population studied, which included a high proportion of newly diagnosed patients and a long follow up.

### Cause-specific mortality

To evaluate cause-specific mortality, ICD-9 and ICD-10 codes were used as reported on death certificates. During the analyses, however, we saw differences in coding practice. One fraction of physicians reported the immediate cause of death, such as ischemic cerebral infarction, while others reported the underlying condition (e.g. atrial fibrillation) leading to an individual’s death. As only one cause of death can be reported in Austria this may lead to underrepresentation of epilepsy and other chronic diseases on death certificates.

Causes of death can be separated into three categories as proposed by Nilsson et al. [29]: (a) underlying disease of which epilepsy is a symptom, (b) underlying disease that has no obvious causal relation to epilepsy, (c) epilepsy that contributes directly to death [29].

Category (a) includes disorders such as congenital anomalies, which were elevated to an SMR of 7.1 (95 % CI 2.3–16.6) overall and 10.8 (95 % CI 1.3–39.3) in the incidence cohort, and thereby slightly lower than previously described at 17.0 (95 % CI 9.5–28.1) by a Swedish cohort [29]. Although not statistically significant due to overlapping confidence intervals, this difference could be explained by selection-bias in their cohort of recruitment of hospital admissions.

The risk for death due to cerebrovascular disease [SMR 2.6 (95 % CI 2.1–3.1), 3.5 (95 % CI 2.6–4.6) for the incidence cohort] was within limits of previous findings where the risk ranged from no elevated risk in patients with idiopathic/cryptogenic epilepsy from England [28] to 2.9–6.3 in other cohorts [28, 29, 31]. Cerebrovascular disease is the most frequent cause of epilepsy in adults over the age of 60. The likelihood of developing early or late epileptic seizures after stroke has been estimated to up to 67 %, for developing post-ischemic-stroke epilepsy to 2–4 % [6]. Less frequently discussed is the occurrence of heraldic seizures, which may have contributed to the number of deaths, attributed to cerebrovascular disease in our study. Heraldic seizures are epileptic seizures caused by hitherto hidden cerebrovascular disease, triggered by clinically silent ischemia occurring before a stroke [7, 15, 32]. In addition, microbleeds frequently seen in conditions such as cerebral amyloidangiopathy or hypertensive angiopathy, might also contribute to the generation of seizures in these patients [8, 15, 21]. Cleary et al. [7] reported an almost threefold increased risk for stroke in patients with onset of seizures after the age of 60 compared to matched controls without seizures. Shinton et al. [32] reported a rate of 4.5 % of patients with a first stroke to have had epileptic seizures previously, and 9.3 % of patients with recurrent strokes developed poststroke epilepsy. The concurrence of focal motor seizures and subsequent side of hemiplegia in stroke in four out of five patients, as well as reduction of seizure severity after stroke in one patient support the hypothesis that seizures may have been caused by silent ischemia in these patients [32].

Malignant neoplasms [SMR 1.2 (95 % CI 1.0–1.4)] were likewise within the range of previously reported findings from not elevated in patients with idiopathic/cryptogenic epilepsy in UK [28] to SMR values of 1.4–5.6 [16, 28, 29, 31] in other cohorts.

In category (b); Causes of death not depicting an etiological cause for epilepsy, might still be associated with the disease, Pneumonia was frequently identified as a cause of premature death in epilepsy patients (SMR 3.5–10.1) [16, 24, 28, 29]. The reason for this is not fully understood. It seems likely that generalized tonic–clonic seizures may lead to aspiration and later pneumonia with lethal consequences. This theory, however, has not been explored up to date. Nevertheless, other pathomechanisms have been discussed. Baumann et al. [4] suggest that neurogenic pulmonary oedema secondary to generalized seizures could be mistaken for pneumonia [28]. Pneumonia is also a common event in the elderly and frail population. Studies of institutionalized patients observed 25 % of patients to die from respiratory death, while this cause was observed in only 5 % of patients in the community [22]. Higher SMR values therefore appear to be due to few cases in younger age groups where pneumonia as a cause of death is rare. This was supported by a British cohort, where pneumonia reached an SMR of 10.3, and the mean age of death was 81 [9]. The Swedish cohort reported an SMR of 4.2 (95 % CI 3.6–4.8) and 40 % of patients were older than 75 years at time of death [29].

The importance of mental health in the epilepsy population has been acknowledged previously as it reduces quality of life, complicates anti-epileptic treatment and increases mortality [5]. In our population mental disorders in general [SMR 2.8 (95 % CI 1.4–4.9), 4.6 (95 % CI 1.5–10.8) for the incidence cohort] and alcohol dependence syndrome in particular [SMR 3.9 (95 % CI 1.8–7.4), 6.4 (95 % CI 1.8–16.5) for the incidence cohort] were significantly elevated causes of death, reflecting the high incidence of severe psychiatric disease and resulting lethal consequences. In a population based study the most common psychiatric co-morbidities in epilepsy patients were depression in 18 %, neuroses in 15 % and psychoses in 9 %, leading to a psychiatric diagnosis in 41 % of patients [14]. Earlier work regarding suicide in epilepsy patients found variable results ranging from no increase [9, 16, 22] to up to a tenfold increased rate [29, 40]. Our data show a clear increase in death from suicide compared to the general population [overall SMR 4.2 (95 % CI 2.0–8.1) and 6.8 (95 % CI 1.4–19.8) in the incidence cohort]. The suicide rate in Austria has steadily declined over the last decade but still remains high compared to other western European countries [11]. Increase in suicide is supported by reports that patients with epilepsy are found to regularly suffer from medically undiagnosed and untreated depression [18] or anxiety [36], which are predisposing factors to suicide [27]. The association of anti-epileptic drug (AED) treatment and suicidal or self-harming behavior has been discussed controversially, but there appears to be a bidirectional association between psychiatric disorders and epilepsy rather than AED treatment [19].

Malignant extracerebral neoplasms in general where only marginally elevated in our cohort at 1.2 (95 % CI 1.0–1.4) and 1.4 (95 % CI 1.0–1.8) for the incidence cohort, whereas is was more significant in other cohorts, especially during the first 5 years of follow-up [28]. An increase of malignancies with long-term use of anti-epileptic drugs was found in some reports [23, 29] but could not be confirmed by others [1, 38]. The finding of reduced immune responses in epilepsy patients, however, could be a possible explanation. Malignancies of the respiratory and intrathoracic organs were elevated to an SMR of 1.5 (95 % CI 1.1–2.0) in our cohort but did not reach significance in the incidence cohort. Likewise, neoplasms of the lung were elevated up to fourfold in a population-based cohort from UK [28]. Other cohorts confirmed this, but had limited validity due to low patient numbers [22, 33]. Explanations for this finding remain speculative. The use of barbiturates has been associated with an increase in lung cancer in one study, but, again, the power of this study was low [13]. In a population-based study from Rochester, USA [16] the rates for cancer were not increased when patients with a cancer diagnosis prior to epilepsy diagnosis where excluded from analysis. Looking at co-morbidities independent of lethality, diseases of the respiratory system were found in 61 % of epilepsy patients, and neoplasms in 7 % [14]. The most common cause for chronic bronchitis and asthma is cigarette smoking [3], which is also a risk factor for cerebrovascular disease and cancer [2], thus can represent an etiological factor for epilepsy itself. Furthermore, epilepsy is more common in lower socioeconomic groups [17], who in return have higher smoker-rates [20]. Another remarkable finding in our cohort was the increase in malignancies of the esophagus with an SMR of 3.1 (95 % CI 1.2–6.4). This was previously investigated only in the Swedish cohort, where a similar increase at 3.3 (95 % CI 2.0–5.2) was observed [29]. Also this type of carcinoma was associated with cigarette smoking and alcohol intake [39]. Surveys have in fact shown an up to twofold higher rate of cigarette smoking among the epilepsy population compared to the general population [25].

Epilepsy itself as a causal factor for death, category (c), could not be fully investigated in this study as information on the occurrence of status epilepticus or potential SUDEP were not available. A twofold increase was seen in external causes of death. Whether this was associated with epileptic seizures could not be evaluated. Though the fact that it was also reported in previous studies [29] supports the assumption of a seizure related cause of death in these patients.

## Conclusions

Epilepsy patients suffer from higher rates of mental health problems as well as increased smoking and alcohol intake compared to the general population [25]. Our study suggests that these conditions may not only result in a poor quality of life, but also in increased mortality. We therefore suggest improving health promotion, including such as cessation of smoking, lowering of alcohol intake and reduction of overweight in patients with epilepsy. Likewise, we see a need for early psychiatric evaluation in the epilepsy population and, if necessary, treatment and support which could carry a significant potential to decrease premature death. Therefore, screening mechanisms should be implemented into every patient’s treatment plan and good mental health be considered a successful outcome parameter for epilepsy treatment. Furthermore, thorough screening for other potential causes of death in epilepsy patients, especially malignancies and cerebrovascular disorders, should be undertaken.

## References

[1] Adelow C, Ahlbom A, Feychting M, Johnsson F, Schwartzbaum J, Tomson T (2006). Epilepsy as a risk factor for cancer. J Neurol Neurosurg Psychiatry.

[2] Barendregt JJ, Bonneux L, van der Maas PJ (1997). The health care costs of smoking. N Engl J Med.

[3] Barnes PJ (2000). Chronic obstructive pulmonary disease. N Engl J Med.

[4] Baumann A, Audibert G, McDonnell J, Mertes PM (2007). Neurogenic pulmonary edema. Acta Anaesthesiol Scand.

[5] Brooks-Kayal AR, Bath KG, Berg AT, Galanopoulou AS, Holmes GL, Jensen FE, Kanner AM, O’Brien TJ, Whittemore VH, Winawer MR, Patel M, Scharfman HE (2013). Issues related to symptomatic and disease-modifying treatments affecting cognitive and neuropsychiatric comorbidities of epilepsy. Epilepsia.

[6] Camilo O, Goldstein LB (2004). Seizures and epilepsy after ischemic stroke. Stroke.

[7] Cleary P, Shorvon S, Tallis R (2004). Late-onset seizures as a predictor of subsequent stroke. Lancet.

[8] Cocito L, Nizzo R, Bisio N, Favale E (1994). Epileptic seizures heralding intracerebral hemorrhage. Stroke.

[9] Cockerell OC, Johnson AL, Sander JW, Hart YM, Goodridge DM, Shorvon SD (1994). Mortality from epilepsy: results from a prospective population-based study. Lancet.

[10] Commission on Classification and Terminology of the International League Against Epilepsy (1989). Proposal for revised classification of epilepsies and epileptic syndromes. Epilepsia.

[11] Eurostat (2014) Death due to suicide, by sex. TPS00122. http://epp.eurostat.ec.europa.eu/

[12] Forsgren L, Hauser WA, Olafsson E, Sander JW, Sillanpaa M, Tomson T (2005). Mortality of epilepsy in developed countries: a review. Epilepsia.

[13] Friedman GD (1981). Barbiturates and lung cancer in humans. J Natl Cancer Inst.

[14] Gaitatzis A, Carroll K, Majeed A, Sander W (2004). The epidemiology of the comorbidity of epilepsy in the general population. Epilepsia.

[15] Garcia-Garcia J, Calleja S, De VV, Salas-Puig J, Lahoz CH (2004). Heraldic seizure. Seizure.

[16] Hauser WA, Annegers JF, Elveback LR (1980). Mortality in patients with epilepsy. Epilepsia.

[17] Heaney DC, MacDonald BK, Everitt A, Stevenson S, Leonardi GS, Wilkinson P, Sander JW (2002). Socioeconomic variation in incidence of epilepsy: prospective community based study in south east England. BMJ.

[18] Hermann BP, Seidenberg M, Bell B (2000). Psychiatric comorbidity in chronic epilepsy: identification, consequences, and treatment of major depression. Epilepsia.

[19] Hesdorffer DC, Ishihara L, Mynepalli L, Webb DJ, Weil J, Hauser WA (2012). Epilepsy, suicidality, and psychiatric disorders: a bidirectional association. Ann Neurol.

[20] Hiscock R, Bauld L, Amos A, Fidler JA, Munafo M (2012). Socioeconomic status and smoking: a review. Ann N Y Acad Sci.

[21] Kiechl S, Furtner M, Knoflach M, Werner P, Willeit J (2007). Kaleidoscopic vision and a jerking leg on the ski slope. Lancet.

[22] Klenerman P, Sander JW, Shorvon SD (1993). Mortality in patients with epilepsy: a study of patients in long term residential care. J Neurol Neurosurg Psychiatry.

[23] Lamminpaa A, Pukkala E, Teppo L, Neuvonen PJ (2002). Cancer incidence among patients using antiepileptic drugs: a long-term follow-up of 28,000 patients. Eur J Clin Pharmacol.

[24] Lindsten H, Nystrom L, Forsgren L (2000). Mortality risk in an adult cohort with a newly diagnosed unprovoked epileptic seizure: a population-based study. Epilepsia.

[25] Lu B, Elliott JO (2012). Beyond seizures and medications: normal activity limitations, social support, and mental health in epilepsy. Epilepsia.

[26] Mohanraj R, Norrie J, Stephen LJ, Kelly K, Hitiris N, Brodie MJ (2006). Mortality in adults with newly diagnosed and chronic epilepsy: a retrospective comparative study. Lancet Neurol.

[27] Morriss R, Kapur N, Byng R (2013). Assessing risk of suicide or self harm in adults. BMJ.

[28] Neligan A, Bell GS, Johnson AL, Goodridge DM, Shorvon SD, Sander JW (2011). The long-term risk of premature mortality in people with epilepsy. Brain.

[29] Nilsson L, Tomson T, Farahmand BY, Diwan V, Persson PG (1997). Cause-specific mortality in epilepsy: a cohort study of more than 9,000 patients once hospitalized for epilepsy. Epilepsia.

[30] Oberaigner W, Stuhlinger W (2005). Record linkage in the Cancer Registry of Tyrol, Austria. Methods Inf Med.

[31] Shackleton DP, Westendorp RG, Trenite DG, Vandenbroucke JP (1999). Mortality in patients with epilepsy: 40 years of follow up in a Dutch cohort study. J Neurol Neurosurg Psychiatry.

[32] Shinton RA, Gill JS, Zezulka AV, Beevers DG (1987). The frequency of epilepsy preceding stroke. Case-control study in 230 patients. Lancet.

[33] Shirts SB, Annegers JF, Hauser WA, Kurland LT (1986). Cancer incidence in a cohort of patients with seizure disorders. J Natl Cancer Inst.

[34] Shorvon SD, Goodridge DM (2013). Longitudinal cohort studies of the prognosis of epilepsy: contribution of the National General Practice Study of Epilepsy and other studies. Brain.

[35] Statistik Austria (2013) Statistisches Jahrbuch Österreichs. ISBN 978-3-902925-12-1

[36] Torta R, Keller R (1999). Behavioral, psychotic, and anxiety disorders in epilepsy: etiology, clinical features, and therapeutic implications. Epilepsia.

[37] Trinka E, Bauer G, Oberaigner W, Ndayisaba JP, Seppi K, Granbichler CA (2013). Cause-specific mortality among patients with epilepsy: results from a 30-year cohort study. Epilepsia.

[38] White SJ, McLean AE, Howland C (1979). Anticonvulsant drugs and cancer. A cohort study in patients with severe epilepsy. Lancet.

[39] Zhang Y (2013). Epidemiology of esophageal cancer. World J Gastroenterol.

[40] Zielinski JJ (1974). Epilepsy and mortality rate and cause of death. Epilepsia.

